# Successes and challenges of implementing teleprehabilitation for onco-surgical candidates and patients’ experience: a retrospective pilot-cohort study

**DOI:** 10.1038/s41598-022-10810-y

**Published:** 2022-04-26

**Authors:** Kenneth Drummond, Genevieve Lambert, Bhagya Tahasildar, Francesco Carli

**Affiliations:** 1 Department of Anaesthesia, McGill University, Montreal, QC Canada; 2 Department of Experimental Surgery, McGill University, Montreal, QC Canada

**Keywords:** Surgical oncology, Oncology

## Abstract

This study documents the implementation of a multimodal teleprehabilitation program (e.g., completion rate, exercise metrics, and program successes and challenges) for cancer patients undergoing surgery. It also documents the patients’ experience of the program. This pilot-cohort study included adults scheduled for elective thoracic and abdominal cancer resection surgery, referred to the prehabilitation clinic to engage in physical activity, and received a teleprehabilitation program between August 1st, 2020, and February 28th, 2021. The technology platform provided to the patients included a tablet and a wearable device to facilitate communication and data collection. Data collected for this article were acquired through virtual physical activity monitoring in addition to patient charts. Qualitative data collected comprised of successes and challenges of implanting a teleprehabilitation program, in addition to patients’ perspectives of the program. Quantitative data collected comprised of the exercise metrics, perioperative functional outcomes, in addition to the surgical and postoperative outcomes. Ten patients (8 males and 2 females; mean age: 68.3 years, SD 11.96) diagnosed with various thoracoabdominal malignancies were included in the current descriptive study. The successes identified were related to recruitment and assessment, improvement in functional capacity, clinic scheduling and interventions, and optimal medical follow-up. The challenges identified were related to the adoption of the technologies by patients and the multidisciplinary team, the accurate acquisition of patient physical activity data, and the initial costs to acquire the new technologies. Patients were satisfied with the teleprehabilitation program (i.e., services delivered; average appreciation: 96%), and they perceived the technologies provided to be 90% user-friendly. The findings of the current study highlight important concepts in view of the current international health paradigm changes prioritizing remote interventions facilitated through digital communication technologies. It provides important insight into the clinical application of telehealth in elderly populations, notably in the context of acute preoperative cancer care. This article may provide guidance for other cancer care facilities aiming to implement teleprehabilitation programs.

## Introduction

The COVID-19 pandemic has had an immense impact on public health, challenging healthcare systems and institutions to adapt to global circumstances and uphold the same quality of care for patients. To this end, many surgeries and medical procedures were delayed indefinitely, prioritizing cases that required urgent interventions, thus creating a backlog of approximately two years for cancer surgerie^1–3^. In addition, the risk of viral exposure continues to be a serious concern for these patients, as they are often immunocompromised, have significant comorbidities and poor lifestyle habits. The implications of preoperative harmful lifestyle modifications, isolation, and quarantine may increase perioperative morbidity, postoperative recovery, and mortality^4–6^.

Prehabilitation aims to improve postoperative outcomes by addressing modifiable risk factors. It has been increasingly recognized to improve functional and clinical trajectories, thereby reducing the burden on both patients and the healthcare system^7^. Prehabilitation interventions are multidisciplinary and typically include an exercise training program, nutritional optimization, psychosocial counseling, pharmacological optimization, glycemic control, and smoking cessation if needed^8^.

In view of the pandemic, remote delivery of prehabilitation services has been suggested^9^ using telehealth platforms paired with technologies (i.e., teleprehabilitation) to address concerns related to cancer patients’ risks, isolation, and deterioration of their health status.

This pilot study aims to document (1) the implementation of a multimodal teleprehabilitation program (e.g., completion rate, exercise metrics, and program successes and challenges) for cancer patients undergoing elective thoracic and abdominal cancer resection surgery, and (2) the patients’ experience of the program.

## Methods

### Study design, ethical approval and patient selection

This retrospective pilot-cohort study describes the delivery of teleprehabilitation programs to onco-surgical patients during the COVID-19 pandemic from August 2020 until February 2021. It was approved by the ethical review board of the McGill University Health Centers (MUHC; Study ID: 2021-7666, approval granted on March 30th, 2021). All methods were carried out in accordance with relevant guidelines and regulations. The inclusion criteria for this cohort study were adult onco-surgical patients scheduled for elective thoracic and abdominal tumor resection. All patients had a good comprehension of the English or French language and agreed to participate in this study by signing a consent form.

### MUHC standard of care during pandemic

Following the onset of the global COVID-19 pandemic in March 2020, hospital access and functions were restricted, and the standards of care and services provided to surgical candidates were negatively affected. Surgical patients were able to be seen in person by nurses, internists, and anesthesiologists in the preoperative clinic. Patients were also asked to perform a precautionary COVID test 24–48 h preceding their surgery to confirm that they were not positive carriers of the virus.

### Referral

Following referral by the MUHC surgeons to the prehabilitation clinic, patients were contacted and offered technology-assisted prehabilitation. Those who accepted were scheduled for an initial assessment at the clinic.

### Clinical evaluation process

**Figure 1:**
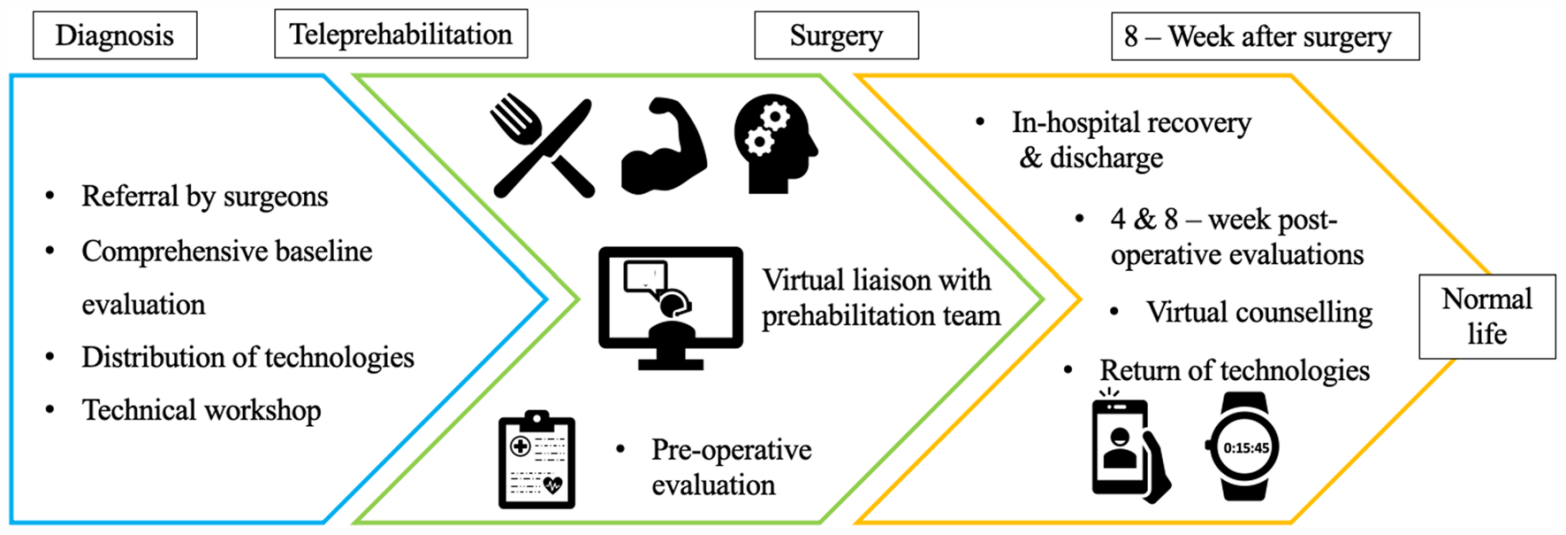
The Patient’s Trajectory through the teleprehabilitation pathway.

The patient’s trajectory from diagnosis through the teleprehabilitation pathway is represented in Fig. 1. Baseline and subsequent evaluations were conducted in the prehabilitation clinic, as permitted by recommendations from the Public Health Authorities and Emergency Measures Coordination Center of the MUHC. To limit unnecessary additional hospital visits, prehabilitation evaluations (baseline, 24 h prior to surgery, and 4 and 8 weeks after surgery) were timed to coincide with patients’ other essential medical appointments. The baseline evaluation was conducted by a physician, an exercise physiologist, a registered dietician, and, if needed, a psychology-trained nurse and lasted approximately 1 hour. During the initial evaluation clinical status (including past medical history, medications, living conditions, and standardized anthropometrics) and functional capacities were assessed using a battery of standardized tests as previously described (6 min walk test, timed-up and go, sit-to-stand, handgrip strength, and 1-arm curl test)^8,10^. Patients were also asked to complete questionnaires to screen for emotional distress (HADS) and malnutrition (PG-SGA), which helped to personalize the interventions to their respective needs.

The figure is a schematic representation of the patient’s trajectory through the teleprehabilitation pathway beginning at diagnosis and ending eight weeks post-surgery. The first phase begins after diagnosis and consists of patient referral by their surgeon, a comprehensive baseline evaluation, provision of technologies, and a related technical workshop. The second phase begins when all the components of the first phase have been completed and is marked by the commencement of the prehabilitation program. The latter includes patient-specific interventions relating to nutrition, exercise (aerobic and resistance exercise), mental health, and medical optimization, in addition to virtual videoconferencing meetings with the prehabilitation team via videoconferencing. A preoperative evaluation is performed 24 h prior to surgery and marks the end of the second phase. The third phase begins after surgery with the in-hospital early recovery, followed by discharge and the continuation of the virtual counseling. During this phase, patients are asked to perform evaluations at 4 and 8 weeks post-surgery. Patients return the technologies to the clinic following the end of their participation in the program. The 8-week post-operative evaluation marks the end of the teleprehabilition pathway, at which point patients should be resuming their normal activities of daily living.

### Prehabilitation program

Following the baseline evaluation, the multidisciplinary clinical team would develop a personalized prehabilitation program for each patient targeting their clinical modifiable risk factors and perceived needs. The program was multimodal and comprised of an exercise program, in addition to nutritional and psychological support. Each patient met in person or virtually with a registered dietician who would review dietary patterns and provide nutritional recommendations. They were also scheduled with a psychology-trained nurse for psychosocial counseling. The exercise physiologist would review the patient's results from the functional test battery and prepare an exercise program tailored to the patient’s functional abilities and clinical objectives. The exercise program comprised of exercise counseling, and aerobic and strength-training exercises. The exercise physiologists followed patients and contacted them weekly for exercise counseling sessions and to monitor their progress, which was possible due to the teleconferencing platform. Patients were educated on the Borg scale (scale ranging from 6 to 20) and asked to aim for a perceived intensity of 12–13 of their preferred aerobic training modality (walking, cycling, swimming). The prescribed duration and frequency were personalized to patients' capacities and schedule with the aim of progressing weekly. The strength training was aimed to be performed twice weekly and included 8 exercises using bodyweight or elastic bands for 2–3 sets and 8–12 repetitions.

### Technologies and material

Following their baseline evaluation patients attended a 45 min in-person technical workshop on the different applications and technologies^11,12^ They were also provided with a training watch paired with either a numerically coded email (generated by the clinic; n = 8) or alternatively their personal email (n = 2). The emails were used to register the technologies with the coded accounts for Polar Flow and Zoom applications. As the latter applications were core to the teleprehabilitation program, the clinic furnished tablets, with predownloaded applications (Zoom and Polar Flow) to facilitate its delivery. The tablets were also equipped with premade educational videos created by the prehabilitation clinic team on nutrition optimization (healthy eating, improving protein and energy intake, portion size, glycemic control) and relaxation exercises (breathing exercise, relaxation, imaging, visualization). Tablets were loaned to seven patients, while three patients were equipped with their personal devices. Beyond the watch and tablet, one patient was also provided with a magnetically braked upright cycle ergometer, which was lent to him for aerobic exercise due to concerns pertaining to knee pain and climate concerns. All patients received complementary material during the initial visit to the prehabilitation clinic: nutritional supplements of whey protein, relaxation recordings, elastic bands (Theraband®), a threshold inspiratory muscle trainer (IMT) device, and a booklet with a personalized exercise prescription.

### Description of the data retrieved

All outcomes were captured via data collected from the Polar Flow application, self-report questionnaires, and patient chart review. Data collected from the Polar Flow application were as follows: the weekly minutes recorded as exercise, the daily steps, the frequency and duration of resistance and the number of aerobic sessions. Self-report questionnaires included a mental well-being questionnaire (Hospital Anxiety and Depression Scale; HADS^13^) completed at each evaluation and a postintervention satisfaction questionnaire (Appendix A). Outcomes obtained from patient charts included neoadjuvant therapies (NAT), emergency department (ED) visits, surgical procedures, length of hospital stay (LOHS), and intra- and postoperative complications. Additionally, the challenges and successes of the program were collected from the patients’ chart and exercise physiologists' clinical notes.

### Outcomes

The primary outcome was the description of the implementation of teleprehabilitation, including the completion rate, intervention-related adverse events, drop-outs and exercise metrics, preoperative functional and clinical trajectories, and program successes and challenges.

The secondary outcome was the patients’ experience with the program presented with qualitative analysis related to the advantages and disadvantages of technology support.

### Data analysis

Descriptive analyses were used for demographics, program exercise metrics, baseline and preoperative functional and clinical outcomes. Means and standard deviations were reported when data were normally distributed; alternatively, medians and ranges were used for parameters with skewed distributions. The distribution of the data was first visually appraised with respect to the interquartile ranges, and in cases of uncertainty, the Shapiro–Wilk test was used to confirm normality^14^.

Additional statistical analyses include a paired t-test comparing means from pre- (T0) and post-tests (T1) performed to assess changes in functional performance and exercise metrics (i.e., first week compared to last week of intervention).

The abovementioned calculations were performed using PASW Statistics software version 24.0, with confidence intervals and significance levels preset at 95% and 0.05, respectively. (SPSS Inc, Chicago, Illinois).

The qualitative analyses were thematic; the quotes were selected from the satisfaction questionnaires and from the exercise physiologists’ clinical notes. The quotes were reported in an anonymized manner relating to two main themes: their satisfaction with the services and their experience with the technologies^15,16^.

### Ethical approval and consent to participate

Following a referral by surgical investigators, the patients were contacted by a member of the research team who discussed the technology-assisted support and expectations of their involvement. If the patient expressed interest, they were invited to visit the prehabilitation clinic at the Montreal General Hospital where informed consent was provided.

## Results

### Patients’ characteristics

Ten onco-surgical patients received teleprehabilitation services, the median age of participants was 68 years old, 8 of whom were male, see Table 1. Patients lived a median distance of 33.75 km (range: 2.7–193.5 km; one-way trip) from the MGH. Details of the cohort’s characteristics can be found in Table 1.

**Table 1:** Demographic and health characteristics.

	Cohort	Missing data
Age, years	68 [52–88]	
Sex-male, n (%)	8 (80%)	
Weight, kg	75.4 (7.4)	
BMI, kg/m^2^	25.5 [22.6–31.1]	
Distance from hospital (km)	33.75 [2.7–193.5]	
**Cancer type, n (%)**		
Colorectal	2	
Lung	3	
Retroperitoneal Sarcoma	3	
Esophagogastric	2	
**Neoadjuvant therapies, n**		
Chemotherapy	3	
Radiotherapy	3	
Chemo-radiotherapy	1	
PG-SGA, score	7 (1–16)	2
Alcohol consumer, n	6	
Current smoker, n	7	
HADS-Anxiety	3 (1–14	1
HADS-Depression	4 (1–11)	1
**Blood biochemistry**		
C-reactive protein, mg/L	7.3 (1.67–130)	1
Albumin, g/L	40.7 (2.4)	1
HbA1c, %	6.16 (0.5)	3
Hemoglobin, g/L	130.2 (22.3)	–
BNP	38 (15–368)	2

Data presented as the mean (SD), median [Range] or n (%); BMI, Body Mass Index; BNP, B-Natriuretic peptide; HADS, Hospital Anxiety and Depression Scale; HbA1c, Glycated hemoglobin; PG-SGA, Patient Generated Subjective Global Assessment; PG-SGA nutritional triage level score of 0–3, an intervention by a dietitian is unnecessary, 4–8, necessitating an intervention by a dietitian and ≥ 9, in critical need for nutrition intervention.

The most common comorbidities were hypertension (n = 8), arthritis (n = 4), cardiovascular disease (n = 4), dyslipidemia (n = 3), diabetes (n = 3), chronic obstructive pulmonary disease (n = 3), and obstructive sleep apnea (n = 2). Other medical conditions reported in the cohort included Guillain-Barré syndrome (n = 1), gastroesophageal reflux disease (n = 1), depression (n = 1), spinal stenosis (n = 1), chronic kidney disease (n = 1), asthma, tinnitus, mild hearing loss and kidney stones (n = 1).

### The program: functional and clinical trajectories, success and challenges

#### Teleprehabilitation program metrics

The median teleprehabilitation period was 9.5 weeks (IQR 2–15) from baseline evaluation and continued throughout neoadjuvant therapies in 9 patients until surgery. One patient did not undergo surgery due to disease progression. No intervention-related adverse events were reported.

During the first week, the average daily step count was 4895 (SD: 1795) and increased by 1358 steps (SD: 1989) by the last week of prehabilitation (*P*-value < 0.001), with six out of ten patients increasing their daily step count. (Table 2).

**Table 2:** Exercise metrics.

ID	Program duration	Daily step count		Weekly exercise [min]		Resistance exercise		Aerobic exercise		Exercise counseling session	
		1st week	Last week	1st week	Last week	Session/Wk	Duration/session	Session/Wk	Duration/session	Total	Duration/session
1	13	6427	6453	65	103	1.08	32	1.85	115	20	27
2	5	5977	7455	194	136	2.4	41	5.4	36.5	14	21
3	2	7480	6303	193	107	2	17.5	4.5	17.5	2	18
4	11	4600	7137	30	451	2.91	32	5.55	53	9	34
5	6	2946	4100	152	229	2	24	3.83	23	12	36
6	12	4693	5994	187	221	0.83	30	4.17	55	12	27
7	15	1161	4500	244	355	2.73	32.5	4.4	33	14	37
8	8	4821	2770	84	169	2.13	43	3.125	50	8	60
9	13	5000	9760	60	200	0.69	37.5	1	32	10	36
10	10	5845	4651	100	120	1.5	30	1.4	30	16	60

ID: patient identifier, 1–10; Program Duration: unit = weeks; Weekly Exercise: data recorded voluntarily as exercise by patient on the polar watch, unit = minutes; Daily Step Count: data recorded by the polar watch, unit: mean step/day; Resistance Exercise and Aerobic Exercise: data recorded voluntarily as exercise by patient on the polar watch. *1st week*: data collected during the patient’s first week of the program; *Last week*: data collected during the patient’s last week before their surgery; *Session/wk*: mean number of sessions per week; *Duration/session*: mean duration per session; unit: minutes; *Total*: total number of sessions through the program supervised by the exercise physiologist.

Although the median weekly time exercising was not significantly different from the Canadian recommendation of at least 150 min of moderate to high-intensity exercise per week, the duration of exercise between the first and last weeks of prehabilitation increased by 58 min (range: − 86 to 421 min; *P*-value: 0.093), with eight out of ten patients increasing their weekly time spent exercising.

#### Preoperative functional capacities

During the preoperative period, no significant increase in 6 MWD was shown, contrarily to TUG and STS (*P*-value: 0.017 and *P*-value: 0.002, respectively; Table 3).

**Table 3:** Preoperative change in functional capacities.

	n	Baseline	n	Preoperative	*P*-value
**Six-minute walking distance**					
Actual, meters^b^	9	426 [210–660]	9	487 [230–603]	0.208
**Timed-up and go,** seconds^b^	9	7.87 [5.47–8.48]	8	5.58 [4.23–8.35]	0.017
**Sit-to-stand, repetitions**^a^	10	11.5 (6.19)	9	12.78 (6.03)	0.002
**Arm-curl test, repetitions**^a^					
Right	8	18 (5.81)	8	18.88 (2.90)	0.186
Left	8	17.88 (6.90)	8	18.5 (2.78)	0.074
**Hand-grip strength, kg**^b^					
Right	10	30 [10–46]	8	30 [12–52]	0.085
Left	10	30 [10–38]	8	30 [12–42]	0.394

^a^Mean (standard deviation), ^b^Median [range].

#### Clinical trajectory

Postoperative recovery was uncomplicated for most patients, with serious complications reported in only three patients (CCI scores: 100, 33.5, and 62.2). One patient was readmitted for drainage of a pelvic abscess. There was one death. (Table 4).

**Table 4:** Surgical and post-operative outcomes.

Surgery	
**Surgical approach**	
Open	4
Minimally invasive	5
**Surgical procedure**	
Lobectomy	2
Esophagectomy	2
Retroperitoneal resection	3
Colectomy	2
**Post-operative recovery and morbidity**	
Patients without complications	5
LOHS	6 [2–47]
Readmissions	1
Reoperations	1
In-hospital mortality	1

Data are presented as n: absolute number of patients, or median [range].

#### Program’s successes

1. *Recruitment and assessment* None of the patients opposed receiving technology-supported prehabilitation interventions. The initial evaluation was either performed at the prehabilitation clinic (n = 7), at the surgeon’s office (n = 2), or while hospitalized (n = 1).
2. *Clinical scheduling and interventions* In the context of the pandemic, evaluations by the exercise physiologist and the dietitian in the clinic were limited to one patient per hour separated by 15 min sanitation breaks. Remote consultations did not impose a burden on in-person clinical functions. The training watches allowed patients to record and self-monitor their training sessions. The exercise physiologists were able to asynchronously monitor patients' patterns of physical activity, pertinent accelerometric, and biometric data objectively. Data collected from the training watch would be synchronized with the application on the tablet daily. The coach profile on the polar website allowed the exercise physiologist to remotely review the physical activity metrics collected from each of the watches. The Zoom teleconferencing application allowed the exercise physiologists to virtually meet with patients, visualize and adapt exercises to the patient’s environment while synchronously providing corrective cues and proper health counseling.
3. *Optimal medical follow-up* All patients were contacted by the exercise physiologist through the Zoom interface at least once per week to revise their weekly physical activity levels, assess patient progress, review the exercises prescribed and modify the prescription as needed. The virtual exercise counseling sessions provided exercise physiologists with the opportunity to inquire about health, symptoms, and general well-being, which was communicated with the multidisciplinary team for the delivery of additional medical (n = 8), nutritional (n = 7), and psychosocial (n = 6) support over phone calls.

#### Program’s challenges

1. *Patient learning curve* Seven patients used the tablets, while all used the polar training watch. Most patients required assistance from a family member (n = 4), caregiver (n = 1) or exercise physiologists (n = 3) to assist them in using the technologies and applications. To this end, 4 patients required continuous assistance throughout the preoperative period, two others required aid only on one occasion (during the first 7–14 days), and the rest did not require any assistance. The three patients who required the exercise physiologist’s help were due to (a) challenges in downloading the applications on their personal devices (n = 1) and (b) performing a factory reset of the device (n = 2).
2. *Physical activity data acquisition* Although patients were trained to actively begin and terminate the recordings and synchronize the watch with the Polar Flow application, patients tended to forget, thus resulting in missed collection of heart rate during the exercise session. All patients, except one, wore the watch regularly (> 4 days/week).
3. *Multidisciplinary team adjustment to new technologies* Only the exercise physiologists and research coordinator communicated with the patient using the Zoom application. Patients received an initial nutritional consultation with a registered dietician either in person (n = 8) or over the phone (n = 3), and all patients received psychosocial counseling sessions over the phone (n = 8), except two who were already being supported by other psychology-trained personnel prior to their recruitment.
4. *Postoperative loss to follow-up* Half of the cohort’s patients were lost to follow-up. This was in part due to disease progression (n = 1), the commencement of adjuvant therapy (n = 1), postoperative complications (n = 1), mortality (n = 1), and discontinued interest (n = 1).
5. *Cost* Patients were lent technologies (Polar Ignite watch, n = 10 and a Samsung Galaxy tablet, n = 7) that facilitate remote real-time counseling and physical activity monitoring. The cost of the tablet was approximately $350, while that of the watch was $250, with an average of $15–$20 per month for patients who required internet data (n = 2). Since the tablets and the watches were reusable, patients were asked to return at the end of the study.

### Patients self-reported experience

#### The program

Eight patients were able to complete a satisfaction questionnaire pertaining to their participation in the program, whereas two patients were unable to complete the questionnaire because of surgical complications (n = 1) and mortality (n = 1). The median satisfaction score for teleprehabilitation was 96% (range: 83 to 100%). The average perceived usefulness score of prehabilitation services was 88% (SD: 10.43). Patients perceived benefits of receiving teleprehabilitation on their physical fitness (n = 8), mental health (n = 6), symptoms of their condition (e.g., pain, nausea, fatigue, etc.; n = 3), social health (n = 2), and diet (n = 1). Other benefits mentioned included “General wellness and support” [Patient #4, #5]. Moreover, one patient attributed her early discharge to her perceived improvements in physical fitness. Quotes from patients are presented in Table 5.

**Table 5:** Quotes from patients.

Theme	n	Quotes
Support	9	“I would not have been able to endure the treatments and the surgery thereafter had it not been for the continuous support I was receiving through the digital platform” [Patient#4]
		“I felt there was somebody on the other side [of the application], the team was reliable […] The exercise physiologist was professional and knowledgeable, and I wouldn’t have completed the program without them” [Patient#1]
		“My son was able to do [the aerobic exercise] with me” [Patient#5]
Inability to enrol in the program if in-person interventions	5	“It takes the tablet. I do not think I could have come [at prehabilitation clinic for the interventions], with the medical appointments and everything” [Patient#5]
		“It would have been impossible due to how far away I live from the hospital” [Patient#2]
Technological literacy	2	“As you know, I have never used these technologies before. I do not own a phone, so it took me some time to learn how to use the tablet. Some of the challenges I had were navigating through the tablet, making/accepting calls via zoom, connecting the audio, and remembering how to synchronize the watch with the app.” [Patient #7]
Recommendation to offer the teleprehabilitation services to other patients	3	“Keep going! You have to do this to get out of the hospital quickly” [Patient#5]
		“I am grateful to have had the support of the [prehabilitation] team during [neoadjuvant] treatments and before surgery” [Patient #7]

#### The technologies

According to the patients, the median score for user-friendliness of the technological system was 90% (range: 53–100%). Three patients mentioned trouble using the tablet and the watch; see Table 6 for the nature of the patient’s challenges with the technologies.

**Table 6:** Patients’ technological challenges.

The tablet	The watch
Mandatory software updates on the tablet	Difficulty connecting the watch to the tablet (through Bluetooth connection)
Prompts to input login information for Polar	Forgetting to wear the watch or to start/stop recording when exercising
Connecting the audio during the video-conferencing meeting	
Locating the messages sent by the prehabilitation healthcare professionals	

## Discussion

This pilot study aimed to document a novel method of delivering prehabilitation services to onco-surgical patients, i.e., teleprehabilitation, and, more specifically, issues related to technology and patients’ experience. The main results showed that telehealth interventions were well received by patients, allowing for greater flexibility in clinical scheduling and exercise interventions. However, challenges remain in its seamless implementation. Notably, hurdles to overcome include the adoption of the technologies by patients and the multidisciplinary team, the difficulty of acquiring accurate data on patient physical activity, and the initial costs of acquiring the new technologies. The patients’ experience also highlighted two aspects of the program: the appreciation for services and the support received and the user-friendliness of the technological system provided.

### Addressing the literature gaps

Telehealth has experienced rapid growth since the COVID-19 pandemic^6,9^, enhancing its utility for diverse clinical applications^17^. Nevertheless, the literature is lacking with respect to the context of interventions and the clinical populations, more specifically with regard to acute care and elderly patients. Even further, there is a lack of consensus in the literature concerning the optimal technological systems for the successful delivery of teleprehabilitation^9^. Two technological approaches have been proposed to overcome technology adoption barriers: 1. readily accessible technologies and 2. combining activity monitoring devices with a secure videoconferencing platform. In the context of the present study, most of the patients were older and had limited access to technologies and the internet.

### The program

Teleprehabilitation is a novel concept, even more so in high-risk surgical cancer patients, prompting the need for increased documentation of its implementation in clinical practice. Two other studies notably documented their delivery of teleprehabilitation programs in this population, both of which were pilot studies with bimodal (exercise and nutrition^18^) and unimodal (exercise-only^19^) interventions. The first study by Bruns et al. provided prehabilitation to frail elderly onco-surgical candidates through a home-based electronic prototype that was created solely for this purpose^18^, while the other study by Piraux et al. used a virtual exercise prescription application and an exercise monitoring watch for esophageal cancer patients^19^. The exercise interventions in both studies were significantly different (daily 7 min prerecorded exercise videos and nutritional recommendations^18^ vs three sessions weekly, including a 30 min aerobic, a 30 min resistance and an IMT training component^19^). Nonetheless, both studies included a weekly phone call to assess adherence^18,19^. Bruns et al. acknowledged that although self-reported adherence was high, the lack of supervision may lead to lower-quality execution and prevent individualization of interventions^13,18^. Furthermore, Piraux et al.^19^ identified that the application interface may not be suitable for patients with lower technological literacy. Nevertheless, patients reported a high overall level of satisfaction with the teleprehabilitation program, and the authors discussed the added value of reducing transportation burden on patients’ schedules^15,16,20^.

In relation to the literature, the current findings reinforce the importance of the appropriate selection of technologies with regard to the simplicity of use (i.e., user-friendly interface) and the possibility of supervising interventions remotely^9^. In all studies, patients were satisfied and comfortable with the technologies, with few patients experiencing minor technical difficulties. Distinctly, the current study included synchronous exercise counseling, which was mentioned to be a limitation of the Fit4SurgeryTV program^18^. Increased supervision has been shown to yield greater adherence rates and improvements in health and functional outcomes^13^. The latter may be attributed in part to increased attention from clinicians who can ensure that patients properly adhere to the exercises prescribed, attaining the appropriate intensity and duration, while applying the necessary modifications and progressions, which aligned with the findings of the current study.

Another novelty of the study was the acquisition of a large variety of exercise metrics from the physical activity watch. Prior to the study, most home-based interventions with the prehabilitation clinic of the MUHC were reliant on patients’ capacity to self-assess their compliance with the programs^21^. Physical activity monitoring devices provide clinicians with important insight into physical activity levels, and unlike phone calls, they offer a more quantitative perspective of movement patterns and behaviours on a day-to-day basis. The technologies create a communication portal between patients and their clinicians, allowing them to better appreciate the patient’s overall exercise volume (i.e., frequency, intensity, time, type). This increased access to information may be helpful in view of adapting and progressing the prescription throughout the preoperative period according to patients’ capacities; however, it brings forth a new challenge for the scientific community to properly quantify and interpret adherence to the program.

### Patient’s experience

The high satisfaction reported in the current study aligns with many telehealth-based interventions in the perioperative field^16,18–20^. Notably, a telerehabilitation by Kairy et al.^16^ aimed to document the patient’s perspective of a telerehabilitation program after hip arthroplasty through interviews. In both studies, patients emphasized their appreciation for the technology’s ease of use and the reduced need for hospital commutes. Moreover, patients in this retrospective cohort reported a sense of accomplishment in being able to positively impact their respective trajectories of care.

### Limitations

While this study showed great clinical potential for the implementation of teleprehabilitation, it is not without its limitations, notably related to its design, evaluations, and interventions. First, due to the design based on the need to describe the methodology, this is an exploratory study with a small cohort with diverse demographics, cancer types, pathologies, and disease management approaches. As such, the data documented in the functional and clinical trajectories section should be seen as contextual information, not presented to draw conclusions. Second, given that some patients were unable to visit the prehabilitation clinic, clinicians performed evaluations in surgical clinics or inpatient units. The latter leads to a minimal functional and demographic assessment, as the exercise physiologist cannot always access the material or space to conduct all tests. Furthermore, several patients did not attend follow-up health assessments due to concerns pertaining to commuting, COVID-19, or conflicts with other medical appointments. Last, a major limitation of the described services lies in the fact that videoconferencing interventions were conducted uniquely by exercise physiologists. Not all the members of the multidisciplinary team were trained or equipped to deliver their services using the new technologies. Future studies should investigate the feasibility and impact of videoconferencing multimodal interventions beyond the scope of exercise.

## Conclusion

This study aimed to document the implementation process of teleprehabilitation in onco-surgical patients after the first wave of the COVID-19 pandemic and report the successes and challenges of this new intervention modality. The successes identified were related to positive acceptance by patients of this technology, convenient clinic scheduling and interventions, and optimal medical follow-up. The challenges observed included the patients’ learning curves, the limited acquisition of physical activity data, the adjustment of the multidisciplinary team to new technologies, the postoperative loss in follow-up, and the initial costs of the technologies. This study further aimed to document the patient’s experience of receiving multimodal teleprehabilitation services. On this subject, patients were satisfied with the teleprehabilitation program (i.e., services delivered), and they perceived the technologies provided to be user-friendly. Future studies should also investigate the feasibility and validity of virtual functional health assessments in the case that visiting the hospital is not a viable option^22^, in addition to investigating ways to improve the documentation of adherence to lifestyle interventions.

## Supplementary Information

Supplementary Information.

## Data Availability

The principal investigator (F.C.) and the research coordinator (B.T.) will have full access to the final database.

## References

[1] Schmunk, R. Catching up on B.C. surgery backlog will take up to 2 years, province says. (2020).

[2] Ross, S. Montreal hospitals backtrack on plan to hire unskilled workers as operating-room help. CTV News Montreal Digital Reporter [Internet]. 2021 Apr 2; Available from: https://montreal.ctvnews.ca/mobile/montreal-hospitals-backtrack-on-plan-to-hire-unskilled-workers-as-operating-room-help-1.5373151

[3] Søgaard M, Thomsen RW, Bossen KS, Sørensen HT, Nørgaard M (2013). The impact of comorbidity on cancer survival: A review. Clin Epidemiol..

[4] Silver JK (2020). Prehabilitation could save lives in a pandemic. BMJ.

[5] Sell, N. M., Silver, J. K., Rando, S., Draviam, A.C., Santa Mina, D., Qadan, M, *et al* Prehabilitation Telemedicine in Neoadjuvant Surgical Oncology Patients During the Novel COVID-19 Coronavirus Pandemic. 10.1097/SLA.0000000000004002 PMC7268857 32675505

[6] Silver JK (2020). Prehabilitation may help mitigate an increase in COVID-19 peri-pandemic surgical morbidity and mortality. Am. J. Phys. Med. Rehabil..

[7] Simcock, R. Principles and guidance for prehabilitation within the management and support of people with cancer, In partnership with Acknowledgements [Internet]. 2019. Available from: https://www.researchgate.net/publication/336617250

[8] Awasthi R, Minnella EM, Ferreira V, Ramanakumar AV, Scheede-Bergdahl C, Carli F (2019). Supervised exercise training with multimodal pre-habilitation leads to earlier functional recovery following colorectal cancer resection. Acta Anaesth. Scand..

[9] Lambert G, Drummond K, Ferreira V, Carli F (2020). Teleprehabilitation during COVID-19 pandemic: The essentials of “what” and “how”. Support. Care Cancer.

[10] Butland RJ, Pang J, Gross ER, Woodcock AA, Geddes DM (1982). Two-, six-, and 12-minute walking tests in respiratory disease. BMJ.

[11] Communication ZV. Zoom.us [Internet]. 2019 [cited 2020 May 8]. Available from: https://zoom.us/pricing

[12] Electro, Oy P. Polar Watch [Internet]. 2019 [cited 2020 May 8]. Available from: https://www.polar.com/ca-en

[13] Stout NL, Baima J, Swisher AK, Winters-Stone KM, Welsh J (2017). A Systematic review of exercise systematic reviews in the cancer literature (2005–2017). PM R.

[14] Ghasemi A, Zahediasl S (2012). Normality tests for statistical analysis: A guide for non-statisticians. Int. J. Endocrinol. Metab..

[15] Lambert G, Alos N, Bernier P, Laverdière C, Drummond K, Dahan-Oliel N (2021). Patient and parent experiences with group telerehabilitation for child survivors of acute lymphoblastic leukemia. Int. J. Environ. Res. Public Health..

[16] Kairy D, Tousignant M, Leclerc N, Côté AM, Levasseur M (2013). The patient’s perspective of in-home telerehabilitation physiotherapy services following total knee arthroplasty. Int. J. Environ. Res. Public Health..

[17] Horsley S, Schock G, Grona SL, Montieth K, Mowat B, Stasiuk K (2019). Use of real-time videoconferencing to deliver physical therapy services: A scoping review of published and emerging evidence. J. Telemed. Telecare..

[18] Bruns ERJ, Argillander TE, Schuijt HJ, Van Duijvendijk P, Van Der Zaag ES, Wassenaar EB (2019). Fit4SurgeryTV at-home prehabilitation for frail older patients planned for colorectal cancer surgery: A pilot study. Am. J. Phys. Med. Rehabil..

[19] Piraux E, Caty G, Reychler G, Forget P, Deswysen Y (2020). Feasibility and preliminary effectiveness of a tele-prehabilitation program in esophagogastric cancer patients. J. Clin. Med..

[20] Doiron-Cadrin P, Kairy D, Vendittoli PA, Lowry V, Poitras S, Desmeules F (2020). Feasibility and preliminary effects of a tele-prehabilitation program and an in-person prehablitation program compared to usual care for total hip or knee arthroplasty candidates: A pilot randomized controlled trial. Disabil. Rehabil..

[21] Bollen JC, Dean SG, Siegert RJ, Howe TE, Goodwin VA (2014). A systematic review of measures of self-reported adherence to unsupervised home-based rehabilitation exercise programmes, and their psychometric properties. BMJ Open.

[22] Blair, C. K., Harding, E., Herman, C., Boyce, T., Demark-Wahnefried, W., Davis, S. *et al* Remote assessment of functional mobility and strength in older cancer survivors: Protocol for a validity and reliability study. Vol. 9, JMIR Research Protocols. JMIR Publications Inc.; (2020). 10.2196/20834 PMC7492978 32769075

