# Supplementary figures and images for: Successes and challenges of implementing teleprehabilitation for onco-surgical candidates and patients’ experience: a retrospective pilot-cohort study
Source: Sci Rep. 2022 Apr 26;12:6775. doi: 10.1038/s41598-022-10810-y (PMC9039599; doi:10.1038/s41598-022-10810-y)

#
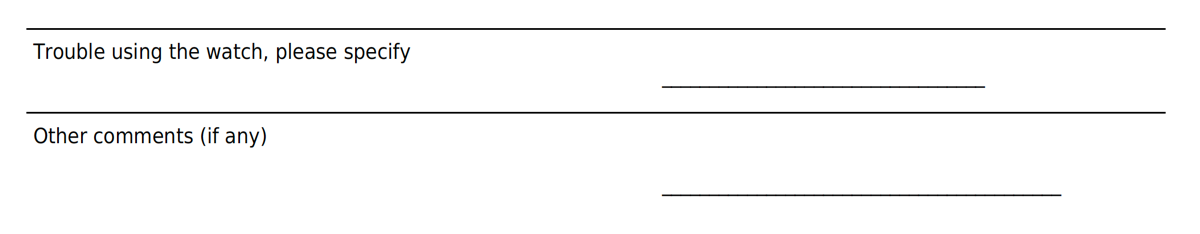
Appendix A: Program Satisfaction Questionnaire

Supplement: Supplementary file 1 — Supplementary Information. [file 41598_2022_10810_MOESM1_ESM.docx]
